# Incidence and cost of perioperative red blood cell transfusion for elective spine fusion in a high-volume center for spine surgery

**DOI:** 10.1186/s12871-018-0591-8

**Published:** 2018-09-05

**Authors:** Giuseppe Ristagno, Simonetta Beluffi, Guido Menasce, Dario Tanzi, Juan C. Pastore, Giuseppe D’Aviri, Federica Belloli, Giorgio Savoia

**Affiliations:** 1 Neurosurgery I Unit, Neuro Center, Humanitas Research Hospital, Via Manzoni 56, 20089 Rozzano, MI Italy; 2 Management Control Unit, Humanitas Research Hospital, Rozzano, Italy

**Keywords:** Spine fusion, Transfusion, Blood, Costs

## Abstract

**Background:**

Spine fusion is a surgical procedure characterized by a significant perioperative bleeding, which often requires red blood cell (RBC) transfusion.

**Methods:**

The incidence and the cost of RBC transfusion were evaluated in all patients undergoing elective surgery for spine fusion in our Institution, a high-volume center for spine surgery, over a period of 3 years. The analysis specifically addressed the RBC transfusion need in all the different spine fusion procedures (atlanto-axial, cervical, dorsal, lumbar, revisions) with the different surgical approaches (anterior, posterior).

**Results:**

During the 3 years of observation, a total of 1.882 elective spine fusions were performed. More than half of the procedures (*n* = 964) were posterior lumbar fusions. Overall, 5% of the patients (*n* = 103) required RBC transfusion. The cervical fusions were the procedures with the lowest percentage of RBC need (0–5%), while the dorsal and the lumbar ones, with the anterior approach, represented the procedures with the highest rate of transfusion (29% and 25% respectively). More than 60 % of the RBC units were employed in the instance of posterior lumbar fusion, while a variable 1–10% of the units was used in each of the other procedures. The overall transfusion cost was of 46.000 euros, with a distribution of costs that paralleled the amount of units transfused for each procedure.

**Conclusions:**

Several surgical and patient factors may contribute to the perioperative blood loss. An accurate patient blood management, may efficiently decrease transfusion requirements and ultimately healthcare costs.

## Background

Spine fusion is a surgical procedure characterized by a significant perioperative blood loss, ranging from more than half to 2 l per case. For this, it has been recognized to be among the top 10 surgical procedures that necessitate red blood cell (RBC) transfusion, with an incidence that can be as high as 30% [1]. However, blood transfusion is known to be associated with a higher risk of adverse events which may contribute to a greater hospital length of stay. Moreover, RBCs have a variable cost per unit transfused that usually ranges between 700 and 1.200 US dollars, plus additional costs of treatment for related side effects [2].

Comprehensive analyses specifically addressing the RBC transfusion need in all the different spine fusion procedures (atlanto-axial, cervical, dorsal, lumbar, revisions) with different surgical approaches (anterior, posterior) are lacking. Thus, this study evaluated the incidence and the cost of RBC transfusion in all patients undergoing surgery for spine fusion over a 3-year period, in a high-volume center for spine procedures.

## Methods

This study was a retrospective cohort investigation, evaluating the incidence and the cost of RBC transfusion in all patients undergoing elective surgery for spine fusion in the Neuro Center, Humanitas Research hospital, Milan, Italy, a high-volume center for spine surgery, over a period of 3 years, between Jan 1st, 2014 and Dec 31st, 2016. The study was approved by the Institutional Review Board. At the Humanitas hospital, all clinical data are prospectively collected and stored electronically in the data management system, in order to be retrieved at any time for clinical and research purposes. At the time of hospital admission, each patient provided informed consent for the use of the data. A retrospective database analysis was performed to identify all patients who underwent spine fusion.

Categorical variables are presented as numbers and proportions. Univariate and multivariate logistic regression was used to identify spine fusion procedures associated with RBC transfusion need. Odds ratios (OR) with the corresponding 95% CI were calculated and *p* values were considered statistically significant if they were less than 0.05. Statistical analyses were performed MedCalc Statistical Software version 17.7.2 (MedCalc Software bvba, Ostend, Belgium).

## Results

During the 3 years of observation, a total of 1.882 elective spine fusions were performed. More than half of the procedures (*n* = 964) were posterior lumbar fusions, while the anterior cervical ones (*n* = 652) covered another one third of the whole cohort (Table 1). Overall, 5% of the patients (*n* = 103) required RBC transfusion. Considering the specific procedures, the cervical ones were those with the lowest percentage of transfusion (0–5%), while the anterior dorsal and lumbar ones accounted for the highest rate, 29% and 25% respectively (Table 1).

**Table 1:** Red blood cell transfusions and costs in the whole 3-year cohort of spine fusion patients

Spine fusion procedure (approach)	All patients n	Patients transfused n (%)	RBC unit transfused Total (average/pt.)	Cost Total (average/pt.), euro	OR for transfusion (OR [95%CI]), *p* value
Atlanto-axial	45	5 (11)	10 (2)	2.180 (436)	2.22 [0.86–5.75], *p* = 0.1
Cervical (anterior)	652	2 (0)	10 (5)	1.756 (878)	0.03 [0.01–0.14], *p* < 0.0001
Cervical (posterior)	55	3 (5)	8 (3)	1.358 (453)	0.97 [0.31–3.25], *p* = 0.99
Dorsal (anterior)	7	2 (29)	8 (4)	1.529 (765)	7.03 [1.35–36.66], *p* = 0.02
Dorsal (posterior)	80	11 (14)	19 (2)	3.960 (360)	2.96 [1.52–5.79], *p* = 0.002
Lumbar (anterior)	8	2 (25)	9 (5)	1.629 (815)	5.85 [1.16–29.36], *p* = 0.03
Lumbar (posterior)	964	69 (7)	158 (2)	28.713 (416)	2.0 [1.32–3.05], *p* = 0.001
Dorsal revision (posterior)	4	1 (25)	2 (2)	362 (362)	5.80 [0.59–56.29], *p* = 0.13
Lumbar revision (posterior)	67	8 (12)	24 (3)	4.164 (521)	2.46 [1.14–5.29], *p* = 0.022
Total	1.882	103 (5)	248 (2)	45.651 (443)	

RBC, red blood cells; pt., patient

With the exception of the atlanto-axial, posterior cervical and dorsal revision spine fusions, all the other spine procedures were significantly associated with the need for transfusion (Table 1). The anterior cervical fusion was, instead, associated with a lesser need for transfusion (OR 0.03, *p* < 0.0001). However, at the multivariate logistic regression, being subjected to an anterior cervical (OR 0.02 [0.01–0.09], *p* < 0.0001) or to a posterior lumbar (OR 0.56 [0.36–0.88] *p* = 0.011) spine fusion was independently associated with a lesser need for transfusion.

**Fig. 1:**
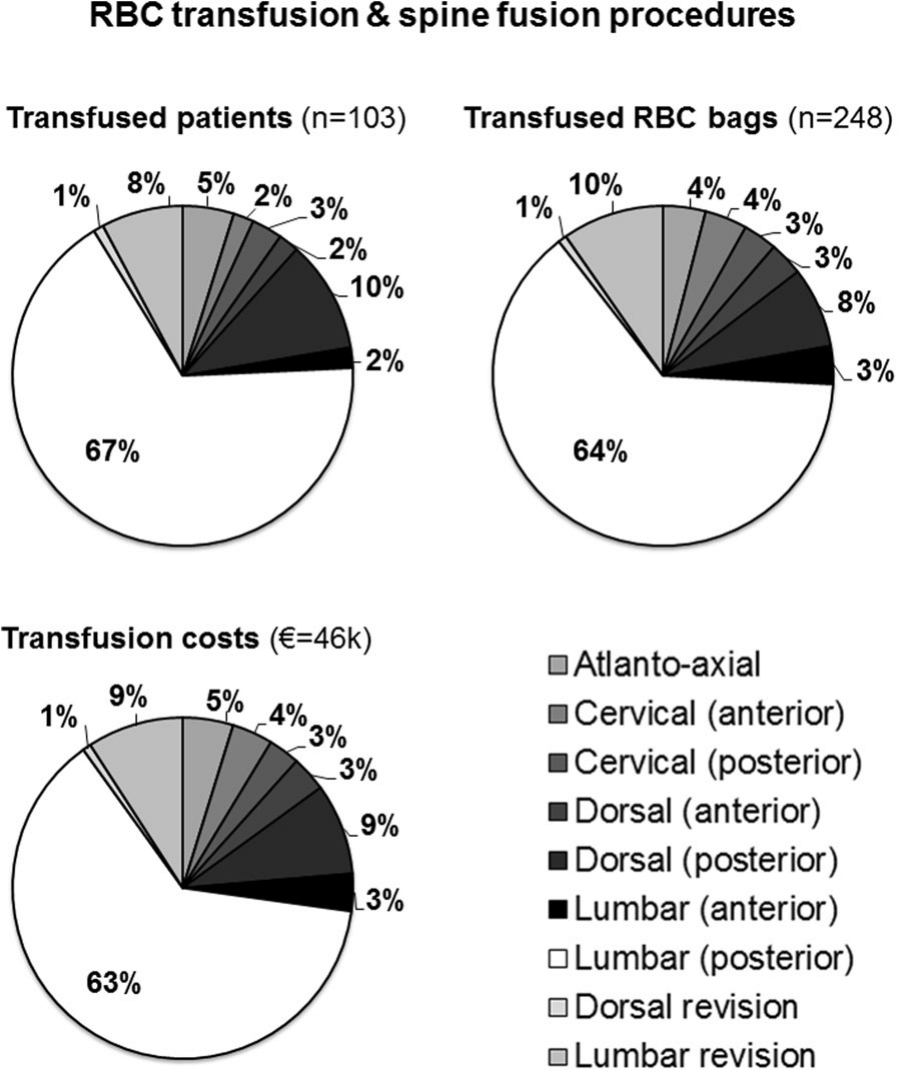
Distribution of patients and red blood cell (RBC) units transfused, together with transfusion costs, in the different spinal fusion procedures

Overall, a total of 248 RBC units were transfused in the 103 patients (Fig. 1). More than 60 % of the units were employed in the instance of posterior lumbar fusion, while a variable 1–10% in each of the other procedures (Fig. 1). The above transfusions accounted for a total expense of almost 46.000 euros, with an average of 443 euro per patient transfused and a distribution of costs that paralleled the amount of units transfused for each procedure, i.e. 63% only for the posterior lumbar fusions.

## Discussion

Several surgical factors may contribute to the perioperative blood loss, i.e. exposure of cancellous bone, stripping of skeletal muscles, extensive spinal instrumentation, and operative time [3]. Indeed, in our database, more complex procedures, i.e. dorsal and lumbar procedures with anterior approaches and revisions of earlier fusions, accounted for a higher rate of transfusion. More than 25% of patients subjected to these procedures received RBCs in the peri-operative, with an OR of 7.03 and 5.85 for dorsal and lumbar anterior fusions, respectively. Although, the overall cost for such highly transfused interventions remained contained due to the low number of cases per year, the cost per patient were almost doubled compared to that recorded for the other fusion procedures, i.e. more than 800 euro vs. 443 euro, and this was related to the fact these transfused patients received approximately 5 RBC units compared to an average of 2 in the other fusions. On the contrary, procedures with a high caseload, i.e. the posterior lumbar fusion, although accounted for the greatest part of overall direct costs, were characterized by a low rate of transfusion (7%) and cost-per patient. Indeed, at the multivariate logistic regression, the procedures with the highest number of cases during the study period, i.e. the anterior cervical (*n* = 652) and the posterior lumbar (*n* = 964) spine fusions, were independently associated with a lesser need for blood transfusion. Likely, such a high rate of spine surgeries per year in our O.U. accounted for a continuous procedure optimization with a concurrent reduction in the percentage of patients needing peri-operative transfusion.

Beside surgery-related factors, patient-specific characteristics, i.e. age, gender, comorbidities, pre-surgical hemoglobin level, have been associated with perioperative transfusion need [4]. Accordingly, these factors were not considered in this investigation since they have been already exhaustively described and indeed previously confirmed in our population of lumbar spine fusions [1, 4]. It has been reported that factors associated with perioperative blood transfusion were older age, female gender, higher ASA grade, presence of diabetes, use of anticoagulant/platelet drugs, longer operative time, and low pre-surgery HB levels [4–7]. An accurate patient blood management strategies, i.e. through pre-operative patient’s preparation (by increasing RBC mass in elder and anemic patients), optimization of surgical procedures, and intraoperative interventions (i.e. antifibrinolytic drugs) have been used efficiently and are advocated to decrease perioperative blood loss and transfusion requirements, and ultimately healthcare costs [8].

## Conclusions

This brief report evaluated the incidence and the cost of peri-operative RBC transfusion in all the different spine fusion procedures with different surgical approaches over a 3-year period in a high-volume center for spine fusion. The anterior dorsal and lumbar fusions and revisions were the procedures characterized by the highest rate of transfusion need, number of RBC units transfused, and transfusion-related cost per patients. Nevertheless, surgical procedures with a greater caseload, i.e. the posterior lumbar fusion, although presented a low rate of transfusion need and number of unit transfused per patient, overall accounted for the greatest part of transfusion costs in spine surgery.

## Abbreviation

RBC: Red blood cell

## References

[1] Yoshihara H, Yoneoka D (2014). Trends in the utilization of blood transfusions in spinal fusion in the United States from 2000 to 2009. Spine.

[2] Shander A, Hofmann A, Ozawa S (2010). Activity-based costs of blood transfusions in surgical patients at four hospitals. Transfusion.

[3] Berenholtz SM, Pronovost PJ, Mullany D (2002). Predictors of transfusion for spinal surgery in Maryland, 1997 to 2000. Transfusion.

[4] Ristagno G, Beluffi S, Tanzi D (2018). Red blood cell transfusion need for elective primary posterior lumbar fusion in a high-volume Center for Spine Surgery. J Clin Med.

[5] Basques BA, Anandasivam NS, Webb ML (2015). Risk factors for blood transfusion with primary posterior lumbar fusion. Spine (Phila Pa 1976).

[6] Morcos MW, Jiang F, McIntosh G, et al. Predictors of blood transfusion in posterior lumbar spinal fusion: a Canadian spine outcome and research network (CSORN) study. Spine (Phila Pa 1976). 2018;(43):E25–39. 10.1097/BRS.0000000000002115 28187072

[7] Lenoir B, Merckx P, Paugam-Burtz C (2009). Individual probability of allogeneic erythrocyte transfusion in elective spine surgery: the predictive model of transfusion in spine surgery. Anesthesiology.

[8] Mehra T, Seifert B, Bravo-Reiter S (2015). Implementation of a patient blood management monitoring and feedback program significantly reduces transfusions and costs. Transfusion.

